# Regulation of mRNA transport, localization and translation in the nervous system of mammals (Review)

**DOI:** 10.3892/ijmm.2014.1629

**Published:** 2014-01-21

**Authors:** CARLO MARIA DI LIEGRO, GABRIELLA SCHIERA, ITALIA DI LIEGRO

**Affiliations:** 1 Department of Biological Chemical and Pharmaceutical Sciences and Technologies (STEBICEF), I-90128 Palermo, Italy; 2 Department of Experimental Biomedicine and Clinical Neurosciences (BIONEC), University of Palermo, I-90127 Palermo, Italy

**Keywords:** post-transcriptional regulation, RNA-binding proteins, neurons, nervous system, synaptic plasticity, RNA metabolism, mRNA pre-localization, non-coding RNA

## Abstract

Post-transcriptional control of mRNA trafficking and metabolism plays a critical role in the actualization and fine tuning of the genetic program of cells, both in development and in differentiated tissues. *Cis*-acting signals, responsible for post-transcriptional regulation, reside in the RNA message itself, usually in untranslated regions, 5′ or 3′ to the coding sequence, and are recognized by *trans*-acting factors: RNA-binding proteins (RBPs) and/or non-coding RNAs (ncRNAs). ncRNAs bind short mRNA sequences usually present in the 3′-untranslated (3′-UTR) region of their target messages. RBPs recognize specific nucleotide sequences and/or secondary/tertiary structures. Most RBPs assemble on mRNA at the moment of transcription and shepherd it to its destination, somehow determining its final fate. Regulation of mRNA localization and metabolism has a particularly important role in the nervous system where local translation of pre-localized mRNAs has been implicated in developing axon and dendrite pathfinding, and in synapse formation. Moreover, activity-dependent mRNA trafficking and local translation may underlie long-lasting changes in synaptic efficacy, responsible for learning and memory. This review focuses on the role of RBPs in neuronal development and plasticity, as well as possible connections between ncRNAs and RBPs.

## 1. Introduction

Post-transcriptional regulation is fundamental for determining the appropriate time and place at which a given mRNA is translated and its half-life. Processes as different as cell fate determination during embryogenesis, differentiated cell response to physiologic or stress cues, and stabilization of memories depend on pre-localization and the regulated translation of specific sets of mRNAs. RNA is thus the carrier of the two messages that are to be translated into the protein and of the detailed information concerning where and whether it is to be translated (1). Moreover, while mRNAs encoding housekeeping proteins, such as cytoskeletal components, have on average short decay rates of days, mRNAs encoding regulatory proteins, such as transcription factors, have half-lives of minutes (2). Deregulation of both short-lived mRNA stability and/or localization/translation may cause marked alterations in cell function, ranging from cancer to cell death.

**Figure 1:**
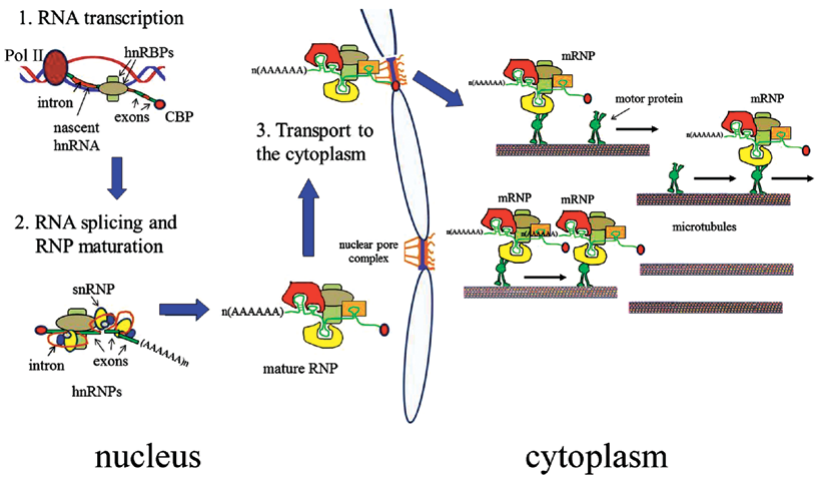
Post-transcriptional fate of mRNA. During transcription (step 1) by RNA Polymerase II (Pol II), the nascent heterogeneous nuclear primary transcript (nascent hnRNA) is complexed with RNA-binding proteins (RBPs) (hnRBPs), some of which are also involved in splicing (step 2), together with small nuclear ribonucleoparticles (snRNPs). In the splicing process, introns are removed and exons are joined together, to obtain a mature mRNA, complexed with RBPs [mature RNA-protein particles (RNP)], ready to be transported to the cytoplasm (step 3), through interaction with proteins of the nuclear pore complex. In the cytoplasm, RNPs attach to cytoplasmic motor proteins, which mediate the microtubule-dependent transport of mRNA to its final destination.

Post-transcriptional regulation largely depends on the activity of a number of general and specific RNA binding proteins (RBPs) that recognize consensus sequences and/or structural features, mainly located in the 3′-untranslated region (3′-UTR) of mRNAs but that can also be present in the 5′-untranslated- (5′-UTR) and coding sequences. Several RBPs bind primary transcripts (heterogeneous nuclear RNAs: hnRNAs) following transcription (Fig. 1), leading to the assembly of nascent heterogeneous nuclear ribonucleoprotein complexes (hnRNPs). In the context of hnRNPs, hnRNA undergoes 3′-end cleavage and the addition of a poly(A) tail, and these events promote transcription termination and the assembly of a dynamic enzymatic complex, the spliceosome. The spliceosome, which contains five small nuclear RNPs (snRNPs) and numerous proteins, is responsible for intron selection and removal, and the appropriate joining of exons (3). RNA protein interactions in the spliceosome are crucial for determining which sequences should be retained in the mature message, and function as checkpoints before mRNA transport to the cytoplasm. Conformational perturbations within the spliceosome can control alternative splicing decisions (4). Notably, the molecular interactions within the spliceosome may be even more complex during maturation of pre-mRNAs containing very large introns, for which nested splicing events have been recently suggested (5), as well as of mRNAs containing ultra-short introns (6). The fact that crucial decisions are taken during maturation of the primary transcript suggests that the nuclear history of mRNA can be determined for its stability and translation into the cytoplasm (7). The nucleo-cytoplasmic traffic of RNPs occurs through the nuclear pore complex (NPC), and requires nuclear export signals (NES), present on RBPs. The directionality of nucleo-cytoplasmic traffic depends on remodelling of the mRNPs on the cytoplasmic side of the nuclear envelope (8). Several RBPs also contain nuclear localization signals (NLS), and shuttle between the nucleus and the cytoplasm. Modulation, by post-translational modifications, of RBP ability to enter the nucleus and/or to exit from it may be one of the earliest regulatory steps in the pathway leading to the translation of a given mRNA.

Once in the cytoplasm, mRNPs are capable of binding to motor proteins, which mediate their transport, in a translationally inactive form, along the cytoskeletal filaments. At their final destinations, mRNAs are immediately translated or stored until a signal-dependent remodelling of mRNPs occurs. Molecular mechanisms allowing the selective translation of pre-localized mRNAs are of the most importance in both the developing and adult nervous system, and can be at the basis of learning and memory processes.

## 2. Regulatory signals and regulatory factors

### General

Post-transcriptional regulation depends on the sequences/structures present on mRNA (*cis*-acting elements), and *trans*-acting factors, and the ability to recognize them. In addition, RNA protein interactions can be modulated over time by extracellular signal transduction pathways that target RBPs, inducing their post-translational remodelling.

### Cis-acting determinants present on mRNA

RNA is a highly dynamic molecule able to fold into complex secondary and tertiary structures, based on standard C:G/A:U base pairs as well as on non-Watson-Crick interactions (9–14) generating different conformers with different biological activities. During folding, RNA has the tendency to be trapped in inactive tridimensional structures (15) and it is not easy to envisage thermodynamically predominant isoforms of a given mRNA (16,17). Two main classes of RNA-binding proteins cooperate *in vivo* with RNA in the acquisition of functional structures: i) RNA chaperones, such as RNA helicases of the DEAD-box family, or unwindases (18–20), which prevent and/or resolve misfolded structures (15,21–23), and ii) tertiary structure-binding proteins, which are able to recognize and stabilize the ‘correctly’ folded RNAs (induced fit) (16).

**Figure 2:**
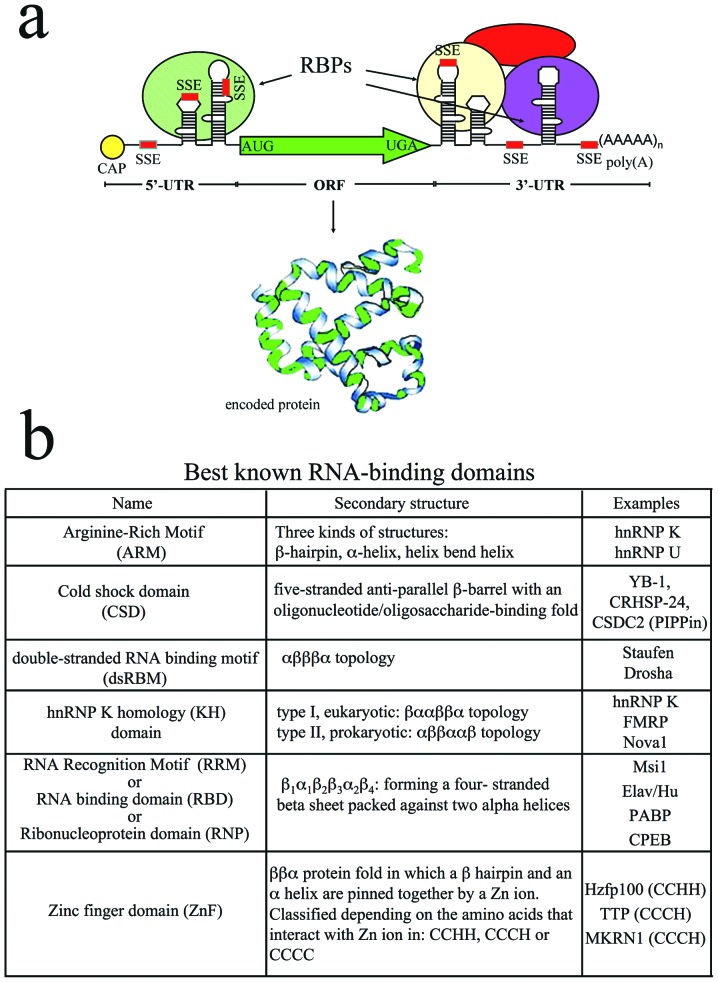
Structural organization of mRNA and best known RNA-binding domains. Schematic representation of (a) mRNA molecule, showing both the open reading frame (ORF, from an AUG codon to a UGA codon) encoding for a protein, and the untranslated regions (UTR) 3′- and 5′- to the coding sequence. The cap structure at the 5′-end, and the poly(A) tail at the 3′-end are also indicated. As described in the text, RNA-binding proteins (RBPs) recognize and bind either secondary/tertiary structures (stem loops in the figure), or simple sequence elements (SSE). The most common RNA-binding domains are summarized in b.

In general terms, *cis*-acting determinants or ‘zipcodes’ can be formed by simple sequences and/or secondary/tertiary structure elements (24,25). Fig. 2a is a schematic drawing of mRNA structural organization. Zipcodes may occasionally function independently of the precise sequence context in which they are normally found: they are capable of driving the localization of any reporter message to which they are fused. However, not all the zipcodes are position-independent. Some RNAs contain multimers of a single motif, while others contain multiple *cis*-acting determinants; in both cases, multiple zipcodes mediate multiple steps in the localization process by acting synergistically (25).

### Trans-acting factors: RNA-binding proteins (RBPs) and non-coding RNAs (ncRNAs)

*Cis*-acting determinants are recognized by RBPs that assemble on RNA, forming RNA protein particles (RNPs) of different and regulated composition. In most cases, *trans*-acting factors recognize a specific secondary structure in the RNA, often a hairpin stem-loop structure, along with a small number of specific anchoring nucleotides (25). RNA recognition and binding is mediated by different families of RNA-binding domains, a summary of which is shown in Fig. 2b.

Most RBPs contain more than one RNA-binding domain, which has been shown to act synergistically by intra-molecular- (26,27) or inter-molecular-cooperativity (28). Conversely, the same RBP is able to recognize different sequence elements within the target mRNA, and recognition can require the correct spatial positioning of these sequences (29). More importantly, binding of all elements can affect overall affinity of the RBP for the target mRNA. This cooperative effect depends on significant unfolding or the structural changes the mRNA undergoes after the first contact with the RBP (26,30). This observation is important when we consider that mRNA also interacts with non-coding RNAs, the regulatory role of which has been recently recognized (see below).

A widely diffused RNA-binding domain is the ribonucleoprotein (RNP) motif or RNA recognition motif (RRM). RRMs contain ~90 amino acids and one or more copies of it were initially identified in proteins that bind heterogeneous nuclear (hn) pre-mRNA (hnRNP) (12,31). Proteins present in hnRNPs also contain a variable number of Arg-Gly-Gly (RGG) repeats that in most cases seem to be non-specific binding domains, acting in cooperation with other RNA-binding domains (31). In alternative or in addition to RRM and RGG motifs, hnRNPs may contain one or more domains known as K-homology (KH) after hnRNP K, the first protein in which the motif was identified (32).

Several RBPs contain an RNA-binding domain with a preference for double-stranded stretches of RNA (dsRNA-binding motif: DSRM). The prototypic DSRM was found in Staufen (Stau), a protein required for maternal mRNA localization in *Drosophila* egg (33). Stau is also involved in the regulation of mRNAs required for memory formation in both *Drosophila* and *Aplysia* (34,35). In mammals, two Stau isoforms are present, which bind to distinct, although overlapping, sets of mRNAs (36). Recently, Stau has been reported to regulate pyramidal cell spine morphology via NMDA receptor-mediated synaptic plasticity (37). Moreover, it probably forms a functional complex with the fragile X mental retardation protein (FMRP) and the 43 kDa transactive response DNA-binding protein (TDP-43), both of which are related to nervous system pathologies (see below) (38).

The most abundant protein family in the mammalian genome is probably constituted by zinc-finger-containing proteins (39), which contain several types of Cys-Cys (CC) or Cys-His (CH) motifs, and were considered primarily as transcriptional regulators. The first identified zinc-finger containing protein, TFIIIA, is necessary for the transcription, by RNA polymerase III, of the genes encoding ribosomal 5S RNA, but is also able to bind ribosomal 5S RNA itself in the 7S ribonucleoparticles stored in the cytoplasm of amphibian oocytes. Similarly, many other members of the family are able to bind the two classes of nucleic acids or RNA only (40,41).

In mammals, one example of CCHH zinc finger domain-containing RBP is hZFP100, involved in the processing of histone mRNAs (42), while a CCCH domain characterizes tristetraprolin (TTP), which participates in mRNA decay (43). TTP and the other members of the TIS11 family have a crucial role in post-transcriptional regulation, targeting for degradation ARE-containing mRNAs (44,45). In rat brain, an interaction between PABP (poly-A binding protein) and Makorin 1 (MKRN 1), a RING zinc finger protein, has been recently demonstrated (46). By binding to PABP and dendritic mRNAs, MKRN1 was able to regulate translation at synapses in response to stimuli inducing synaptic plasticity (46).

Another domain, found in RBPs as well as in DNA-binding proteins, is the cold-shock domain (CSD), first identified in bacterial RNA chaperones and then found in a number of eukaryotic proteins (Y-box proteins), which is able to interact with single-stranded DNA and/or RNA (47). In one of the best studied CSD-containing proteins, the Xenopus protein FRGY2, the CSD was shown to be important for the sequence-specific RNA-binding, while a second tail domain was involved in translation repression (48). Y-box protein (YB)-1, the prototypic member of the CSD-containing protein family, is both a transcription factor and a major component of mRNPs. We recently demonstrated that in nuclear extracts from rat brain, YB-1 interacts with a group of proteins that bind the mRNA encoding the histone variant H1°. Among these proteins another CSD-containing protein, CSD-C2, also known as PIPPin (49), was present (50).

Proteins that do not contain any conventional RNA-binding domains have been shown to bind RNA. The variety of *trans*-acting domains should therefore be larger than expected. Examples of proteins, already known for other well-established functions, which are also able to bind RNA, include thymidylate synthase (51), mitochondrial glutamate dehydrogenase (52), cytosolic glyceraldehyde-3-p dehydrogenase (53), and the calmodulin-binding protein PEP-19 (also known as PCP-4 in humans) (54).

Besides RNA-binding proteins, regulation of the mRNA metabolism also involves non-coding RNAs (ncRNAs). A high proportion of complex genomes gives rise to ncRNAs, among which the most widely studied class is that of miRNAs, small RNAs of ~22 nucleotides (nt) that recognize specific sequences present in the 3′-UTR of target mRNAs. By binding their target mRNAs, miRNAs mediate post-transcriptional gene silencing, through an increase of mRNA decay rate and/or inhibition of translation (55–58). Deregulation of miRNA functions is involved in cancer development (59–61) and in a number of other human diseases, including neurological syndromes (62).

Biogenesis of miRNA is a multi-step process that starts in the nucleus with the transcription of a long precursor (pri-miRNA). Following the sequential action of the RNase III enzymes Drosha and DICER, a dsRNA of ~22 nt is produced in the cytoplasm, resulting in the formation of the RNA-induced silencing complex (RISC) in which only one strand of the dsRNA is inserted that contains members of the Argonaute (Ago) protein subfamily as catalytic endonuclease components (56).

An increasing number of functional interactions have been identified between RBPs and miRNAs. In general terms, RBPs and miRNAs may cooperate or counteract in the regulation of a given mRNA. Examples of the two types of RBPs-miRNA interplay have been identified in different types of cancer cells (63). The role of miRNAs in neurons has also been identified and previously assessed (64,65,66). Since a number of RBPs are uniquely, or almost uniquely, expressed in the nervous system, the possibility exists that they are capable of regulating the interaction among mRNAs and certain groups of miRNAs in a specific way, in turn regulating the establishment and/or maintenance of the unusual functions of this tissue.

### Signaling pathways that control mRNA localization and metabolism

**Figure 3:**
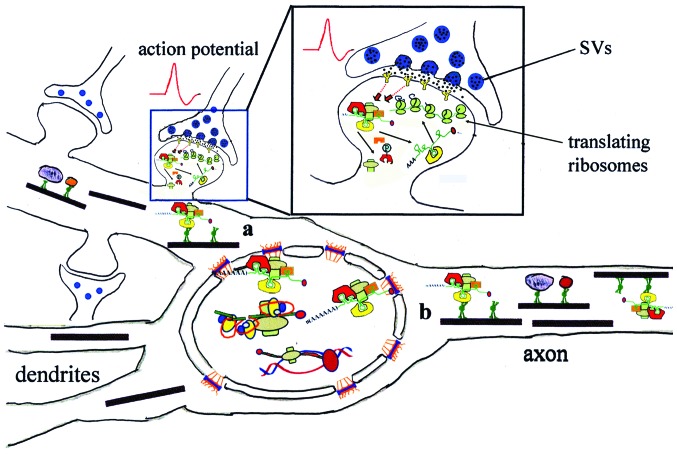
RNA traffic in neurons. As shown in Fig. 1, at the time of transcription, nascent RNA was already complexed with RNA-binding proteins (RBPs) to form RNA-protein particles (RNPs), which then migrate to the cytoplasm through the nuclear pore complex. In the cytoplasm, through interaction with motor proteins, RNPs are transported to their final destinations, where they can pause in a silent state, until they are activated by a signal. Some components of RNPs can then be cycled back to the nucleus. When exiting the nucleus, however, two different peripheral destinations are available to RNPs: a) dendrites or b) axon. Localized translation of mRNA may be controlled by synaptic activity (action potential). In response to neurotransmitters and/or neuromodulators, which bind to their receptors on the post-synaptic element, signal transduction pathways can be activated (red dotted lines), which target RBPs, inducing post-translational modifications, such as phosphorylation. RBP modifications in turn cause remodelling of the RNPs, with the release of some RBPs and recruitment of mRNA to ribosomes. Newly synthesized proteins accumulate at the post-synaptic sites, thus inducing long-lasting modifications. SVs, neurotransmitter-containing synaptic vesicles. Synthesis, maturation, and transport of RNPs are shown as in Fig. 1.

All the steps of RNA metabolism, from splicing and maturation in the nucleus to final degradation, are affected by the cellular microenvironment and by extracellular signals, such as hormones, growth factors, physical and chemical stress stimuli, as well as neurotransmitters (Fig. 3). All these cues are known to affect mRNA stability and translation by triggering signaling pathways that cause post-translational modifications of specific RBPs. A variety of post-translational modifications affecting RBPs have been identified, including serine/threonine phosphorylation, proline hydroxylation, arginine/lysine methylation, lysine ubiquitination or SUMOylation and lipidation. These chemical modifications influence affinity and/or specificity of protein-protein and/or RNA-protein interactions, and are consequently critical for the rapid remodelling of ribonucleoprotein complexes and for the stability and localization of mRNAs (2,67).

Localized mRNAs are transported close to the plasma membrane or organelles, suggesting that membrane-bound kinases play an important role in the regulation of the translation of localized transcripts, in response to extracellular signals (68). Extracellular cues also activate retrograde pathways that may even reach the nucleus, inducing the modification of chromatin organization of genes and, in turn, gene expression. In this manner, responses to extracellular signals and neural activity may also be converted into a stable modification of the cell phenotype.

At least three major mechanisms are known for dynamic modification of chromatin organization: i) chromatin remodelling by ATP-dependent complexes (69); ii) post-translational modification of histone proteins (70), and iii) replacement of histone isotypes present in chromatin with other isotypes (71). During rat brain development, at least histones H3.3, a core histone variant, and H1°, a linker histone variant, have been shown to accumulate during the terminal differentiation of nerve cells (72,73), in the absence of gene activity changes. Post-transcriptional regulation of the expression of these two histones probably depends on the activity of a group of RNA-binding proteins (74,49), some of which have been identified and cloned (75,76). Among these proteins, one was already known as Purkinje cell-expressed peptide (PEP-19, or PCP-4 in humans), but is also expressed in a neuron-specific manner in other brain regions (77,78).

PEP-19 is a calmodulin-binding protein that is known to bind H1° mRNA (54). Similarly, PIPPin (also known as CSDC2) binds H3.3 and H1° mRNAs (49); it is a phosphoprotein that can be phosphorylated by different kinases (79, unpublished data), and that interacts with other proteins already known to bind RNA (50). It may therefore be hypothesized that, in response to calcium-dependent neuronal activity, proteins of this type are modified. Their post-translational modification may in turn change their interaction with histone variant mRNAs, thus inducing their translation and accumulation of the corresponding proteins. Histone variants could finally enter chromatin, thus modifying the structural organization and expression of specific genes.

## 3. Messenger RNA trafficking and localization in neurons

Mature transcripts can be localized through at least three mechanisms: i) local protection from degradation, ii) diffusion and local anchoring, and iii) direct transport by interaction with the cytoskeleton and cytoskeleton-associated motor proteins (80–82). Subcellular pre-localization of mRNAs and locally regulated translation offer cells at least three important advantages: i) energy saving: a high number of protein molecules can be obtained locally by transporting and translating a single mRNA molecule; ii) efficacy/safety of protein production: some proteins may be harmful to the cells when synthesized in the incorrect location; the myelin basic protein (MBP), a major component of the axon-wrapping myelin sheet, produced in the central nervous system (CNS) by oligodendrocytes, is, for example, a sticky protein that potentially interacts with any cell membrane if produced in the cell body (12,83,84); other proteins, such as actin, tubulin and microtubule-associated proteins, exist as different isoforms, all able to form multimers/polymers; their localized synthesis allows the formation of only the right multimers; iii) differential translation regulation: in response to local signaling, proteins may be synthesized only in the compartment exposed to the signal (85,86).

Pre-localization of mRNA is determined in polarized cells (87). The most asymmetric cells in the body are neurons, which consist of a cell body or soma, several branched dendrites and a long axon that can be 1,000-fold the diameter of the cell body (88). The most noteworthy property of nerve cells is the variety of morphologically different sub-compartments. Therefore, how a neuron, with only one cell nucleus and thus a single supply of active genes, obtains such a motley distribution of proteins if of interest and should be investigated.

### mRNA pre-localization in dendrites

Through utilization of different experimental approaches, such as *in situ* hybridization, and amplification of mRNAs present in neuronal processes, the presence in dendrites of a large number of mRNAs was clearly demonstrated. Among the dendritically localized messages the most intensively studied are those encoding microtubule-associated protein 2 (MAP2), the brain-derived neurotrophic factor, the α subunit of the calcium/calmodulin-dependent protein kinase II (CaMKIIα), the activity-regulated cytoskeleton-associated protein (Arc), the NMDAR NR1 subunit, and the AMPA receptor (84,89,90). Dendritic RNA transport is specific and can be regulated by neuronal activity in a rapid manner (91,92).

Information required for the successful mRNA transport to dendrites is sometimes contained in a single zipcode, as in the case of the non-protein coding, dendritically localized BC1 mRNA, which contains, at its 5′-end, a 62 nt zipcode, able to fold into a single stem-loop and to drive microtubule-dependent transport (25,93–95). Other mRNAs have more complex localization signals. The MAP2 mRNA, for example, contains a 640 nt zipcode element in its 3′-UTR, probably created with distinct sub-elements that fold into multiple stem-loops (96), and are able to mediate individual steps of RNP assembly and localization process. Similarly, the mRNA encoding the myelin basic protein in oligodendrocytes contains two distinct elements: i) A2RE, an 11 nt element found only in transcripts that are localized (97); it forms the binding site for hnRNP A2 and is necessary and sufficient for transport (98,99), and ii) a 1 kb long element, essential for appropriate localization of a protein-coding reporter RNA, and probably involved in RNA anchoring (25). Of note, the A2RE element was also identified in the neuronal mRNAs encoding CaMKIIα, Arc and neurogranin, all of which were assembled in the same granules containing hnRNP A2, and targeted to dendrites (100). Conversely, it has also been reported that the dendritically localized mRNAs encoding MAP2, CaMKIIα, and β-actin assemble in distinct RNPs, which contain very few RNA molecules (101,102).

Besides positively acting elements, elements that inhibit transport also exist. Localization of CaMKIIα depends on the 3′-UTR (103), which contains three positive *cis*-acting regions: i) a stretch of ~1200 nt, in the latter half of the 3′-UTR, sufficient for localization (104); ii) one element present in the distal 170 nt of the UTR, which contains cytoplasmic polyadenylation elements (CPEs) (105), and requires a wild-type CPE-binding protein (CPEB); iii) a third zipcode, within the first 94 nt of the 3′-UTR, which contains the already mentioned, 30-nt long sequence with high homology to an element present in neurogranin mRNA. Other elements in the CaMKIIα mRNA, on the contrary, inhibit its transport, in the resting state, while inhibition is eliminated in the depolarized neurons as activity-dependent derepression is critical for their dendritic targeting (106).

Taken together, these findings suggest that assembly and transport of mRNAs involves different signals and highly specific RNA scanning by regulatory proteins. This complexity is likely at the origin of the several types of RNPs that have been described, such as transport RNPs, stress granules (SGs), processing bodies (P-bodies), and P-body-like structures (107–112). Composition of the complexes may vary with growth conditions, and different mRNA subsets are potentially present in different classes of particles; conversely, the same mRNA species may be found in multiple mRNP complexes. Based on these observations, Keene and Tenenbaum suggested that RBPs may regulate mRNAs as subpopulations during cell growth and development. Their model predicted that functionally related genes were regulated as groups or post-transcriptional operons, by specific mRNA-binding proteins that recognize sequence elements common among the mRNAs (113). One of these ‘master’ RBPs that controls the expression of several mRNAs at a time is the Fragile X mental retardation protein (FMRP), which regulates dendritic mRNA transport and local protein synthesis on the post-synaptic side of synapses. Over 400 mRNAs have been reported to associate with FMRP. FMRP interacts with kinesins and is able to repress translation of its client mRNAs both *in vitro* and *in vivo*. More recently, FMRP has also been found in axons and growth cones, where it seems to be involved in responses induced by Semaphorin-3A (Sema3A) (114).

The specific interaction between RNAs and RBPs suggests the existence of an ‘*RNA signature*’ that characterizes each transcript (84,100). For example, the BC1 RNA assembles with proteins to form an RNP involved in the transport of dendritic mRNAs (93); the signal responsible for its dendritic targeting is a motif at its 5′-end, which folds into a single stem-loop instead of into a cloverleaf structure, as expected on the basis of its sequence similarity to tRNA. Notably, tRNAs also migrate to dendrites, but some of them remain and function in the cell body. It has been suggested that its structural properties allow BC1 RNA to be transported to dendrites more efficiently than tRNAs (94).

Further sources of *cis*-acting signal variability are alternative splicing and alternative polyadenylation site selection. These processes are capable of generating different RNA isoforms with different targeting specificities. In brain neurons, two distinct pools of brain-derived neurotrophic factor (BDNF) transcripts are produced by the differential use of polyadenylation sites, i.e., mRNA molecules with the longer 3′-UTR, but not those with the shorter one, contain localization elements, which target them to dendrites (115). At the same time, the longer 3′-UTR also mediates BDNF translation repression in the resting state and activity-dependent translation (116). In a recent study, it has been found that the longer 3′-UTR contains an AU-rich element (ARE) that interacts with the neuronal RBP HuD. Such an interaction is necessary and sufficient for the stabilization of the longer mRNA isoform (117).

A shorter and longer 3′-UTR species also exists in the case of CaMKIIα mRNA, although the long mRNA is much more abundant. Again, the sequence required for dendritic targeting has been mapped downstream to the first polyadenylation site (104), suggesting that the shorter mRNA isoform is confined to the cell body, while the longer mRNA is transported to dendrites. It therefore seems that the strategy of alternative 3′-UTRs may be commonly adopted by genes encoding proteins with important functions in the cell body and dendrites.

As mentioned, *cis*-acting elements interact with RBPs that, by recognizing and binding them, control mRNA localization and stability and thus play an important role in the development and maintenance of the nervous system. Moreover, coordinated expression of neural genes may be obtained by assembly of their mRNAs in common mRNP complexes, which can contain different RBPs under different cell states and at different times (118). This process is of particular importance in modulating synaptic plasticity which seems to depend on the activity-induced translation of hundreds of locally targeted mRNAs, present in neuronal processes, where they drive a variety of specific functions (119).

For instance, mammalian zipcode-binding protein 1 (ZBP1) is required for the dendritic targeting of β-actin mRNA both in developing and mature neurons (120–122). Knockdown of ZBP1 in cultured hippocampal neurons reduced the dendritic levels of ZBP1 and β-actin mRNA and impaired the growth of dendritic filopodia in response to BDNF treatment (120). This observation is important when considering that the formation and density of dendritic filopodia during neuronal development are likely to be connected to the process of synaptogenesis (120,123–125). Phosphorylation of ZBP1, in response to extracellular signals such as BDNF, by the membrane-bound Src tyrosine kinase results in a decreased affinity of ZBP1 for its bound mRNA and consequent local activation of mRNA translation (126–128). However, only ~50% of β-actin mRNA colocalizes with ZBP1 (92), thus suggesting the involvement of other RBPs in the localization/translation regulation of this mRNA. Another RBP required is the Src-associated with a mitosis of 68 kDa (Sam68): knocking down Sam68 in neuronal cultures or interfering with its binding to β-actin mRNA causes a deficit of β-actin mRNA in dendrites and in spine density (122), while loss of Sam68 has been connected with the pathogenesis of neurodegenerative fragile X tremor/ataxia syndrome (FXTAS) (129,130).

The zipcode-binding protein, ZBP2, was identified in chicken embryo brain by RNA affinity chromatography (through binding to an RNA fragment containing the zipcode) (131). ZBP2 is a predominantly nuclear protein that also affects β-actin mRNA localization in the cytoplasm; it is the chicken homologue of the human KH domain-containing splicing regulatory protein (KSRP), a protein involved in pre-mRNA splicing (132). The homologue of ZBP2 in rat is the MAP2-RNA *trans*-acting protein (MARTA1), which binds the 3′-UTR of MAP2 mRNA, a dendritically localized neuronal transcript (133,134). ZBP2 has been found to bind the nascent β-actin zipcode co-transcriptionally and to facilitate binding of ZBP1 to the zipcode (135). It provides an example of how interactions between RNA-binding proteins and RNA serve to recruit and stabilize additional proteins to form a large RNP.

Another well-studied factor involved in mRNA localization and, probably, modulation of translation is the already mentioned HnRNP A2 which binds MBP mRNA, allowing its correct localization in the oligodendrocyte processes in which myelination occurs (127). hnRNP F has been found to be a component of MBP transport granules, and to cooperate with HnRNP A2 in regulating MBP expression. Activity of this factor is controlled through phosphorylation by the Fyn kinase (136). HnRNP A2 was found in dendrites, in association with other hnRNPs, in large macromolecular complexes (neuronal transport granules) that contain mRNA, pre-mRNA splicing factors, and mRNA export factors (137). The presence of proteins with different roles in RBP granules is a common observation: for example, the complex responsible for dendritic targeting of a CaMKII reporter contains PSF (polypyrimidine tract-binding protein-associated splicing factor), hnRNPU and Staufen 1 RNA-binding proteins (127).

### mRNA pre-localization in axons

When discussing mRNA localization determinants in neurons, a challenging issue comes from the finding that mRNAs can be also localized in axons. For a long time, this possibility was not considered since the translational machinery did not seem to be present at significant levels in axons. Moreover, mechanisms ensuring protein transport from cell bodies to the axonal compartment were, on the contrary, present and efficient, thus making apparently unnecessary localized protein synthesis. However, findings of previous studies have demonstrated axonal localization and translation of several mRNA (138–144), including the β-actin mRNA (145–147), which depends on the zipcode recognized by ZBP1 for localization (148).

Together with β-actin transcript, mRNAs for which axonal localization has been reported include those for oxytocin and vasopressin (149,150), β-tubulin (151), actin-depolymerizing factor ADF/cofilin (152), the microtubule-associated proteins 1b (MAP1b) (153) and τ (154), the cytoskeleton regulating protein RhoA (155), the κ opioid receptor (156) and heat shock proteins (157). Notably, among axonally localized mRNAs, transcripts encoding transcription factors have been found, an example being CREB mRNA (158). Moreover, after injury, the population of localized mRNA enlarges to include many transcripts encoding proteins of the translational apparatus and trans-membrane receptors (159). Thus, as with dendrites, a supply of mRNAs exists in axons, and some of these transcripts can be translated under special circumstances (160). In addition, the pool of axonal mRNAs markedly changes during development: in embryonic axons exclusively transcripts belonging to the ‘cellular assembly and organization category’ were found. These transcripts can be further subdivided into smaller subsets of cytoskeletal-related mRNAs and transport of vesicles/trafficking-related mRNAs (161). These observations suggest that, at least in the growing axons, localized protein synthesis may allow dynamic remodelling of the axons during their progression through the extracellular environment.

Localization and translation of mRNA in axons still pose two main problems. The first one concerns the organization and regulation of the translational machinery: axoplasm structural domains have been recently described (periaxoplasmic ribosomal plaques: PARPs) which contain ribosomes attached to a superficial plaque-like structural matrix, together with β-actin mRNA, ZBP-1, SRP54, myosin Va and kinesin II molecular motor proteins. Rapid axoplasmic transport of microinjected heterologous radiolabeled BC1 RNA to putative PARP domains suggested that the anchored translation machinery potentially represents the destination of specifically sorted mRNAs (162). Moreover, multiple translation components, including ribosomal subunits and initiation factors, interact with the trans-membrane receptor (DCC) for netrin-1, suggesting that their activity can be regulated by extracellular signals (163). The possibility that ribosomes/mRNAs could be at least in part horizontally transferred into axons from surrounding glial cells was also discussed (142,164). In addition, it has been reported that proximal segments of transected sciatic nerves accumulate newly synthesized RNA in axons, and that these mRNAs are actually synthesized in Schwann cells and then transferred to neurons through a mechanism that requires actin cytoskeleton and myosin-Va (165).

The second problem concerns specific transport to dendrites and/or axons for those mRNAs that use at least some identical zipcodes for both localizations. Preferential localization depends on the entire supply of RBPs assembled in a given RNP, including those necessary for anchoring RNPs to the cytoskeleton. Several aspects of microtubule organization, among which the specific microtubule-associated proteins (MAPs) and motor proteins, are different between axons and dendrites. These differences may be at the origin of specific sorting of cargoes and their differential targeting in neurons (143,166).

## 4. Locally regulated stabilization and/or translation of pre-localized mRNAs in neurogenesis

### General

Neurogenesis is an extremely complex, multi-step process through which self-renewing undifferentiated neural stem cells obtain different and integrated differentiated phenotypes (167). This process also occurs in the adult nervous system following ischemic insult or damage (168,169), and has been implicated in learning, neuronal plasticity and memory formation in hippocampus (170,171).

### Regulation of mRNA metabolism during development

During development, neurons sprout processes or growth cones, which explore their environment and guide pathfinding over extremely long distances. It is now clear that at least some aspects of axon guidance require axonal mRNA translation (138–140,142–147). For example, Welshhans and Bassell developed and used a new *in vitro* turning assay allowing these authors to demonstrate that growth cones exhibit protein synthesis-dependent attraction to netrin-1 and BDNF. This attraction is lost in neurons lacking ZBP1; concomitantly, BDNF-induced β-actin mRNA localization is also attenuated. ZBP1 is also necessary for netrin-1-induced local translation of β-actin mRNA (148).

Responses of growth cone to guidance cues such as netrin-1, BDNF, and Sema3A proceed through cycles of desensitization and resensitization that are critical for navigation over large distances. Notably, in isolated growth cones, the resensitization step is eliminated by treatment with translation inhibitors, while the sensitization step is affected by inhibitors of endocytosis, but not by translation inhibitors (160,172,173). Thus, only some steps of growth cone response to guidance cues require translation; when required, however, the activation of translation is triggered by signal transduction pathways which involve different kinases, including phosphatidylinositol-3-kinase (PI3K), mammalian target of rapamycin (mTOR), and mitogen-activated kinases (MAPKs) (160,174).

On the other hand, in the absence of induction signals, as well as during the translocation to mRNA final destinations, translation is inhibited. Some RBPs, such as ZBP1, block translation initiation by inhibiting recruitment of the 60S subunit of the ribosome. In other cases, RBPs modulate the length of poly(A) tail (174,175). Conversely, the cytoplasmic polyadenylation element-binding protein (CPEB) binds its recognition element (CPE) in the 3′-UTR of its target mRNAs and promotes polyadenylation-induced translation (176). Block of CPEB function in hippocampal neurons reduces the translation of β-catenin, induced in the growth cones by neurotrophin 3 (NT3), an effect that is probably mediated by calcium ions and CaMKII (177).

Translational regulation by RBPs can also be influenced by miRNAs; these latter molecules are capable of binding RBPs which are, in turn, able to regulate their abundance (65,174,178–180). Several miRNAs are developmentally regulated in mammalian neurons and have been identified in axons (181,182) and dendrites (65).

RBPs are expressed in region-specific patterns in developing brain, suggesting that they are crucial in the establishment of cell type-specific functions (174,183). For example, the splicing regulator Rbfox1 (A2BP1), which may involved in the control of neuronal excitation and seizures, in the mammalian brain (184), is differentially expressed at different developmental stages and in different brain regions (185). In cortical neurons, A2BP1 accumulates in the nucleus, the cell body and dendrites, while in cultured hippocampal neurons it is mainly present in the nucleus but also evidenced in the proximity of synapses (185).

During cell division, asymmetric segregation of RBPs into one daughter cell also contributes to specify its fate, and to generate a cell lineage. For instance, it has been recently demonstrated that the RNA-binding protein Stau2 is asymmetrically distributed during progenitor divisions in the developing mouse cortex, thus determining the asymmetrical distribution of the mRNAs to which it binds (186,187). In the radial glial precursor, Stau2 forms a complex with other RBPs, such as Pumilio 2 (Pum2) and DEAD box 1 (DDX1), and with a number of mRNAs, including those encoding β-actin, mammalian prospero (prox1) (187), and the E3 ubiquitin ligase tripartite motif protein 32 (Trim32) (186). Perturbation of the complex induces premature differentiation of the radial glial precursors into neurons and mislocalization of the target mRNAs (187). Thus, it seems that asymmetric localization of RBPs can regulate the balance of stem cell maintenance versus cell differentiation, as well as cell fate.

Musashi-1 (Msi1) RBP was first reported to play a role in the development of the *Drosophila* adult external sensory organ (188). In mammals, Msi1 is considered a specific marker for neural/progenitor stem cells (189–193). It acts by suppressing translation of mRNAs encoding differentiation-inducing proteins, such as the membrane protein Numb which is involved in the Notch/Delta signalling cascade (118,191,194,195), and the cyclin-dependent kinase inhibitor p21^WAF-1^ (196).

The embryonic lethal abnormal vision (ELAV)-like proteins form a highly conserved RNA-binding protein family, functionally involved in the stabilization of mRNAs which bear AU-rich elements (ARE). As with their *Drosophila* homologue, neuronal-specific proteins ELAV (nELAVL, originally HuB, HuC, and HuD) are necessary and sufficient for inducing neuronal differentiation in mammalian neural precursors (197–199) and are therefore considered markers of post-mitotic neurons (200). The nELAVL proteins were discovered through studies on paraneoplastic neurologic disorders (PND) (201). Some common tumors, such as small-cell lung, ovarian and breast cancers, produce proteins normally expressed in the nervous system; thus, the antitumor immune response is also directed against nerve cells, triggering neurologic symptoms (202). A number of reports have demonstrated that the overexpression of ELAVL proteins induces the stabilization of ARE-containing mRNAs. Ratti and colleagues found that nELAV proteins colocalize with, and bind to, Msi1 mRNA, in the adult mouse subventricular zone and in cultured neural stem/progenitor cells. They suggested that Msi1 and nELAV RNA-binding activities might be complementary and exert a different function on their target mRNAs. In other words, nELAV stabilization of the Msi1 transcript may prolong its expression during the gradual passage from proliferation to differentiation. This would allow the stem/progenitor cell to continue to divide even after Msi1 transcriptional inactivation (118). The post-transcriptional regulatory action of nELAV proteins on Msi1 mRNA seems to be evolutionarily restricted to mammals, and active from embryonic to adult neurogenesis. In adult neurogenic areas, nELAV activity might be transiently induced by spatial- and/or temporal-restricted signals. It has also been suggested that upregulation and cytoskeletal translocation of nELAV proteins, and the subsequent positive effects on Gap43 mRNA levels may be involved in synaptic plasticity and learning processes (see below), in the rat dentate gyrus (203,204).

As mentioned, nELAV proteins are considered early markers of neuronal commitment and are expressed in a specific temporal order in the developing nervous system of vertebrates. In the adult mouse, specific patterns of expression of each nELAV member have been reported in different neuronal types, both in the central and peripheral nervous systems, thus suggesting a role of nELAV proteins in the maintenance of various types of postmitotic neurons: for instance, HuC is strongly expressed in all neurons of neocortex, while HuD is prevalent in the large projection cells of the internal pyramidal layer, and HuB is detectable only in scattered neurons (205,206).

Of the target mRNAs regulated by the nELAV proteins some, in turn, encode proteins involved in mRNA metabolism, including mRNA which encodes the neuro-oncological ventral antigen 1 (Nova1) (207), a neuron-specific splicing factor that controls several mRNAs important for synaptic function by alternative maturation (208,209). Nova1 mRNA stability and translation are positively controlled by nELAV proteins. Moreover, PKC-dependent nELAV phosphorylation induces the recruitment of Nova1 mRNA to polysomes (207). These findings suggest that, as in the case of transcription factors, a hierarchy of RNA-binding proteins exists whose members are expressed as part of a regulatory cascade.

### Regulation of mRNA metabolism in learning and memory

The most noteworthy implication of locally regulated mRNA translation is its possible involvement in long-term changes that accompany the complex processes of learning and memory. Although Hu proteins were originally identified as early markers of neuronal differentiation (210), persistence of HuB, HuC and HuD mRNAs in the adult nervous system, especially in the hippocampus and neocortex, indicates that they are also involved in adult neuronal plasticity and spatial learning (200,204,211).

In mammals, HuD can form dimers and trimers that associate with bound mRNAs, with cytoskeleton proteins and with other ELAV-like proteins to form complexes (204) which, in turn, may associate with polysomes to form translationally competent complexes involved in learning (206) and in long-term memory storage and recall. The parallel increase of cytoskeleton proteins and HuD in the CA1 hippocampal region suggests a common mechanism of regulation.

A learning-specific increase of rodent nELAV proteins was demonstrated in both cell bodies and proximal dendrites of hippocampal pyramidal cells. Notably, the concomitant upregulation of HuD and GAP-43 expression, together with co-localization of HuD and GAP-43 mRNA, have been described in hippocampal pyramidal cells only in rodents that had learned two different spatial discrimination paradigms (204,206). An effect of HuD in learning and memory has also been suggested by using human HuD-overexpressing mice, which do not show any motor/coordination deficits or apparent developmental abnormalities (212). Although HuD expression increases after learning, constitutive overexpression of this protein leads to deficits in behavioural tests such as contextual fear conditioning (CFC) and Morris water maze. These results suggest that HuD levels are tightly regulated and any alteration results in abnormal mRNA stabilization and cognitive impairment (212). Moreover, HuD is probably involved in the stabilization of GAP-43 in growth cones, where granules containing HuD also colocalize with GAP-43 mRNA and ribosomes present in these regions (213). HuD was found to be associated *in vivo* with large cytoplasmic granules in the neuronal cell body and smaller granules in dendrites. Both types of granules were also stained with the ribosomal marker Y10B, suggesting that they also contain ribosomes (203).

The nELAV proteins seem to represent the final target of a cellular cascade that involves PKC, and induces the downstream stabilization of specific mRNAs, implicated in memory trace formation (206). The role of PKC was confirmed in cultured hippocampal neurons, where phosphorylation of HuD regulates BDNF, NGF, NT-3 and GAP-43 mRNAs stability and protein expression. HuD was suggested to be negatively regulated by CARM1 methyl transferase, whose activity is controlled by PKC-dependent phosphorylation (214).

Besides nELAV proteins, which have been the most extensively studied RBPs, a few other proteins are involved in learning and memory. Iijima and colleagues, for instance, identified Hematopoietic Zinc Finger (Hzf), an RBP which regulates the dendritic localization of mRNAs in neuronal cells. This protein is highly expressed in Purkinje cells and binds to the 3′-UTR of type 1 inositol 1,4,5-trisphosphate receptor (IP3R1) mRNA. Post-transcriptional regulation by Hzf affects synaptic plasticity and motor learning in cerebellum, because Hzf^−/−^ mice exhibit severe impairments in motor coordination and motor learning related to cerebellar functions (215,216).

Obviously, the importance of RBP expression is paralleled by the importance of RNA *cis*-acting elements recognized by these proteins. Mutations in the 3′-UTR of mouse CamKIIα mRNA cause *in vivo* the disruption of dendritic localization and translation of CamKIIα, specifically affecting long-term memory formation, suggesting that CamKIIα may be a component of the translation system necessary for memory consolidation (217).

## 5. RBPs linked to human disease

Considering their critical functions in development, it is not surprising that altered RNA protein interactions lead to severe pathologies, such as neurodegenerative diseases and complex neurologic syndromes. Fragile X syndrome (FXS), the most common form of inherited mental retardation, depends on mutation of a gene, located in the X chromosome, which encodes the already mentioned RNA-binding protein FMRP. Most clinical cases of FXS result from hyper-expansion and methylation of CGG repeats within the promoter of fmr1 that cause deficit of its expression. FMRP is involved in the transport and translational regulation of several dendrite-localized mRNAs, including that encoding Arc (218,219), a protein required for hippocampal long-term depression (LTD) and long-term potentiation (LTP) (220), and the amyloid precursor protein (APP) (221,222), thus FMRP function is related to long-term synaptic plasticity. FMRP and its target mRNAs form granule-like complexes that are transported with a kinesin-dependent mechanism along microtubules (223).

Mice bearing a ko-fmr1 gene show specific defects in trace fear memory and LTP loss in the anterior cingulate cortex and lateral amygdala, while retaining normal locomotion capacity and pain sensitivity (224). The interaction between fmr1 and mGluR5, a Gp1 glutamate receptor, whose increased activity is one of the consequences of FMRP loss of function, was studied in mice bearing a ko version of fmr1, crossed with mice which showed a reduced mGluR5 expression. The results revealed that, in animals with 50% reduction of the activity of mGluR5, the symptoms of FXS were reduced, counterbalancing the defects caused by fmr1 mutation (225). The neuronal hyperexcitability linked to a lack of FMRP, which generates epileptogenic responses, resembles the effects of a lack of the already mentioned BC1 RNA, suggesting that BC RNAs and FMRP have similar and overlapping modes of action in translational repression at synapses (226).

FMRP deficiency was reported to affect the proliferation rate in cultured adult neural progenitor/stem cells (aNPCs), isolated from ko-fmr1 mice. Moreover, mutated aNPCs have a higher tendency to differentiate into astrocytes as compared to aNPCs derived from normal brains. Notably, in mutated aNPCs, the Wnt signaling pathway as a whole is altered, and this alteration can be at the origin of the observed modification of cell differentiation balance (227). *In vivo*, deletion of FMRP from aNSCs leads to deficits in learning tasks that depend on adult hippocampal neurogenesis (228). Using ko-fmr1 mice, a marked impairment of cognitive functions was demonstrated, accompanied by a decrease of post-synaptic proteins, and in particular the NMDA receptor, although the relationship between the two events has not yet been established (229).

Alterations similar to those observed in FMRP-mutated mice were also found in translin knockout mice. Similar to FMRP, translin is an RNA-binding protein that is able to associate with microtubules and motor proteins, and is involved in the transport and translation of mRNA localized in dendrites (230). Similarly, huntingtin (Htt) and the Htt-associated protein 1 (HAP1) have been reported to be part of dendritic large transport granules which also contain microtubule-dependent motors and transport β-actin mRNA (109).

Another degenerative pathology that may involve altered RNA-protein interactions is frontotemporal lobar degeneration (FTLD). FTLD has been classified into two subtypes: i) the first one is characterized by accumulation of the microtubule-associated protein τ (FTLD-τ) and ii) the second one is characterized by inclusion bodies that do not contain τ, but contain ubiquitin (FTLD-U) (231). Recently, a major component of FTLD-U inclusion bodies has been identified as TAR DNA-binding protein-43 (TDP-43), a protein of 43 amino acids encoded on chromosome 1, that can bind both DNA and RNA and functions as a splicing factor. Besides FTLD, amyotrophic lateral sclerosis (ALS) and other related neurodegenerative disorders show inclusions of TDP-43 and are now known as TDP-43 proteinopathies (232). TDP-43 extracted from tissues of FTLD or ALS is often hyperphosphorylated, ubiquitinated and poorly soluble (231). Of note, several mutations in ALS have been found in the gene encoding angiogenin (ANG), a secreted RNase (233). Angiogenin and TDP-43 have been identified in stress granules, assemblies of different RBPs and mRNAs. Moreover, angiogenin stimulates the transcription of rRNA and is responsible for the production of stress-induced small RNAs (234). As mentioned in a previous section, TDP-43 has also been found in ribonucleoprotein complexes, which also contain Stau and FMRP (38).

As a final example, proximal spinal muscular atrophy (SMA) is an autosomal recessive disease caused by alteration of the Smn1 gene, that encodes SMN, a protein present in the cells as part of a complex which also contains Sm proteins and U snRNAs, the core components of the pre-mRNA splicing machinery. Recently, it has been reported that KH-type splicing regulatory protein (KSRP), a multifunctional protein closely related to chick ZBP2 and rat MARTA 1, and involved in the exosome-mediated degradation of ARE-containing mRNAs, interacts with SMN. This interaction is lost in the naturally occurring mutations of SMN, found in Type 1 SMA patients (235). Another partner of SMN is the ribonucleoprotein-R (hnRNPR), a protein involved in the axonal transport of β-actin mRNA. Loss of β-actin mRNA in axonal growth cones of hnRNPR-depleted motor neurons resembles the condition observed in SMN-deficient motor neurons (236).

## 6. Conclusion and future directions

As reviewed in the previous sections, a number of studies have previously clearly demonstrated the importance of mRNA localization and the regulated translation in neuronal functions ranging from axon pathfinding in development to nerve regeneration in the adult nervous system, as well as in the long-lasting changes involved in learning and memory.

However, a number of issues remain to be resolved. It is, for example, still uncertain whether and how mRNAs are actually anchored in the active sites of cell processes, e.g., whether silent RNPs are stably associated with specific synapses. An interesting model has been proposed by Doyle and Kiebler (84), who suggested that RNPs containing the mRNAs necessary, for example, for specialized dendritic functions are not strictly anchored in a single site but, instead, continuously move, patrolling groups of synapses in dendrites. RNPs should then be specifically captured and modified in order to allow translation of the cargo mRNAs only from sites in which synaptic activity is high. They named their model ‘*sushi belt model*’, after the conveyor belt used in most Japanese sushi restaurants: the belt conveys sushi to all the potential consumers; however, the hungriest ones consume more items. In the same manner, synapses recently activated should be ‘hungrier’ than silent ones and are likely to capture/modify RNPs (84).

The most puzzling question is how a given mRNA is specifically delivered to dendrites (pathway ‘a’ in Fig. 3) or to the axon (pathway ‘b’ in Fig. 3). The microtubule-associated proteins MAP2 and τ are enriched in dendrites and axons, respectively; however, it remains to be determined whether these and/or other cytoskeleton-associated proteins have a role in selecting RNPs. As mRNAs already associate with some RBPs in the nucleus, whether RNA final destination have been decided from the beginning remains to be clarified, and if this occurs specificity factors should be found among nuclear proteins.

It seems that glial cells are involved in regulating localized translation in axons, through the horizontal transfer of ribosomes (142). An attractive working hypothesis can thus be that glial cells also transfer into neurons regulatory factors, including transcription factors and RBPs, by sorting them to membrane microvesicles and/or to exosomes, which are then shed from glial cells and fuse with neuronal plasma membranes, delivering their cargoes into nerve cells.

It also remains to be defined how miRNA and RBPs cooperate in regulating mRNA metabolism in neurons. In these latter cells, ncRNAs may have an additional role in mRNA silencing during transport and storage processes. In conclusion, mRNAs, miRNAs and RBPs form dynamic complexes in which part of the components probably have only structural functions, while others confer upon mRNA the ability to be specifically sorted to a given sub-cellular compartment and/or to be translated in response to extracellular signals. In other words, RNPs may be regarded as ‘mobile nucleosomes’, possibly originating from an ancient RNA world.
